# Total Neoadjuvant Therapy Versus Long-Course Chemoradiotherapy in Locally Advanced Rectal Cancer: Real-World Tumor Response and Clinical Outcomes

**DOI:** 10.3390/medsci14030393

**Published:** 2026-07-14

**Authors:** Sorinel Lunca, Wee Liam Ong, Stefan Morarasu, Ana Maria Musina, Cristian Ene Roata, Raluca Zaharia, Gabriel Mihail Dimofte

**Affiliations:** 1 Grigore T Popa University of Medicine and Pharmacy, 700115 Iasi, Romania; sorinel.lunca@umfiasi.ro (S.L.); william05021990@gmail.com (W.L.O.); ana-maria.musina@umfiasi.ro (A.M.M.); cristian.roata@umfiasi.ro (C.E.R.); raluca.zaharia11@yahoo.com (R.Z.); mihail.dimofte@umfiasi.ro (G.M.D.); 2 2nd Department of Surgical Oncology, Regional Institute of Oncology, 700483 Iasi, Romania

**Keywords:** locally advanced rectal cancer, total neoadjuvant therapy, long-course chemoradiotherapy, clinical complete response, pathological complete response, magnetic resonance imaging, sphincter preservation

## Abstract

**Background:** Total neoadjuvant therapy is becoming a preferred option for locally advanced rectal cancer, particularly in patients with high-risk baseline features. However, real-world evidence comparing tumor response, MRI-defined high-risk feature clearance, surgical outcomes, and survival after total neoadjuvant therapy versus conventional long-course chemoradiotherapy remains limited. This study aimed to compare outcomes between total neoadjuvant therapy and long-course chemoradiotherapy in patients with locally advanced rectal cancer treated in routine clinical practice. **Methods:** This is a retrospective, single-centre cohort study focused on patients with stage II–III locally advanced rectal adenocarcinoma treated with curative-intent neoadjuvant therapy using either total neoadjuvant therapy or long-course chemoradiotherapy. Tumor response was assessed using restaging MRI, clinical complete response, and pathological complete response. Surgical outcomes and overall survival were evaluated. **Results:** A total of 110 patients were included. Patients treated with total neoadjuvant therapy had a higher baseline disease burden reflected by a greater proportion of cT4 tumors (40.6% vs. 19.2%; *p* = 0.014). Radiologic tumor-length response and clearance of MRI-defined high-risk features were comparable between treatment strategies. Clinical and pathological complete response rates were numerically higher in the total neoadjuvant therapy group, but the differences were not significant (cCR: 15.6% vs. 6.4%, *p* = 0.151; pCR: 18.5% vs. 9.7%, *p* = 0.301). **Conclusions:** In this real-world cohort, TNT was preferentially used in patients with more advanced baseline disease and showed numerically higher complete response rates, although differences were not statistically significant. Radiologic response, surgical outcomes, and short-term survival were comparable between treatment strategies. These findings support the feasibility of TNT in routine clinical practice but should be interpreted as exploratory and hypothesis-generating rather than evidence of treatment superiority.

## 1. Introduction

The management of locally advanced rectal cancer (LARC; cT3–4 and/or node-positive disease) has evolved substantially over the past several decades. Total mesorectal excision (TME) remains the cornerstone of curative treatment, particularly when a negative circumferential resection margin (CRM) can be achieved. CRM involvement is a well-established predictor of local recurrence and poor long-term oncological outcomes, underscoring the importance of effective preoperative treatment strategies aimed at tumor downstaging and margin clearance [1,2,3].

For nearly two decades, preoperative long-course chemoradiotherapy (LCCRT) followed by TME has been considered the standard treatment for rectal cancer. This approach improves local disease control, increases rates of tumor downstaging, and enhances the likelihood of achieving margin-negative resections compared with surgery alone [4,5,6,7].

Total neoadjuvant therapy (TNT) has recently emerged as an alternative treatment paradigm. In contrast to conventional treatment sequencing, in which systemic chemotherapy is partially administered postoperatively, TNT delivers the entire course of systemic chemotherapy before surgery. Several randomized trials have demonstrated that TNT increases rates of pathological complete response (pCR) and clinical complete response (cCR), improves compliance with systemic therapy, and may reduce the risk of distant metastasis compared to classic chemoradiotherapy-based strategies [8,9,10]. More recent systematic reviews and meta-analyses have further supported the oncologic advantages of TNT compared with conventional neoadjuvant chemoradiotherapy [11,12,13,14,15]. Furthermore, the higher clinical and pathological complete response rates observed with TNT have generated increasing interest in organ preservation strategies, particularly in patients who achieve a sustained clinical complete response and may therefore be candidates for non-operative “watch-and-wait” management [16,17,18,19].

Advances in high-resolution magnetic resonance imaging (MRI) have also improved risk stratification in rectal cancer. MRI enables the identification of radiological high-risk features, including tumors or lymph nodes in close proximity to the mesorectal fascia (MRF+) and the presence of extramural venous invasion (EMVI+) [20,21,22]. These features are strongly associated with an increased risk of local recurrence and distant metastasis [2,21,22,23,24]. Consequently, patients with such high-risk radiological characteristics may derive particular benefit from intensified neoadjuvant treatment strategies such as TNT [8,9,16,18].

Although TNT has proven a solid option in RCTs [25,26,27,28], these results may not fully reflect real-world clinical practice, where treatment decisions are influenced by patient comorbidities, tumor location, performance status, and institutional treatment pathways. Multidisciplinary decision-making is therefore essential, and treatment outcomes in routine clinical settings may differ from those reported in controlled trial populations [25,26,27].

However, evidence comparing TNT and LCCRT in routine clinical practice remains limited, particularly with respect to combined clinical, pathological, surgical, survival, and MRI-defined response endpoints. This gap is clinically relevant because real-world treatment allocation is influenced by baseline tumor risk, comorbidities, performance status, and multidisciplinary decision-making, which may differ substantially from randomized trial settings. Therefore, this study aimed to compare tumor response and clinical outcomes after TNT versus standard LCCRT in patients with LARC treated in routine clinical practice. In addition, we evaluated MRI-based tumor response and high-risk feature clearance, while accounting for baseline differences between treatment groups using propensity score matching and adjusted regression analyses.

## 2. Materials and Methods

### 2.1. Study Design and Patient Selection

This retrospective single-center cohort study was conducted at the Regional Institute of Oncology, Iași, Romania, and included patients treated between 1 January 2019, and 31 December 2025. The study protocol was approved by the Institutional Ethics Committee of IRO Iași, Romania, approval number 179, on 30 March 2026. Given the retrospective design and use of anonymized clinical data, the requirement for informed consent was waived.

Patients were identified through the institutional hospital discharge database. A total of 2368 discharge records with a principal diagnosis of rectal cancer were retrieved. After consolidating repeated hospitalizations and removing duplicates, 810 individual patients were screened. Demographic, clinical, imaging, treatment-related, surgical, pathological, and survival data were collected from institutional medical records.

Patients were eligible if they had histologically confirmed rectal adenocarcinoma, clinical stage II–III locally advanced rectal cancer, curative-intent neoadjuvant treatment with either TNT or LCCRT, and available baseline clinical and MRI staging data. For patients who underwent surgery, resection had to be performed at our institution to ensure consistency of surgical technique and pathological assessment.

**Figure 1:**
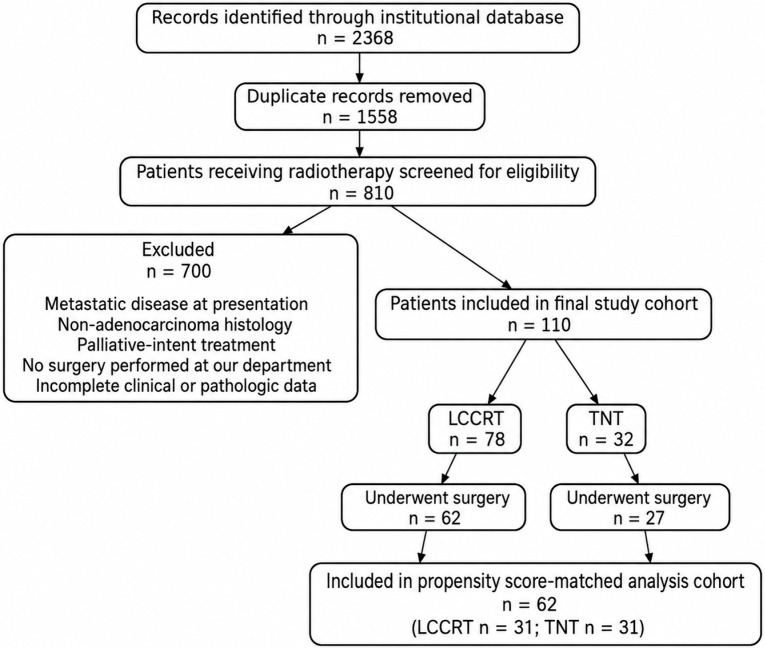
STROBE flow diagram of patient selection and analytic cohorts.

Exclusion criteria were palliative-intent treatment, non-adenocarcinoma histology, synchronous malignancy, metastatic disease at presentation, and surgical resection outside our department. After applying these criteria, 110 patients were included, including 78 treated with LCCRT and 32 treated with TNT (Figure 1).

### 2.2. Staging and Restaging

Baseline staging and post-neoadjuvant restaging were performed using high-resolution pelvic MRI according to standardized rectal cancer imaging protocols, including high-resolution T2-weighted sequences in axial, sagittal, and coronal planes aligned with the tumor axis; diffusion-weighted imaging sequences were routinely acquired. Restaging MRI was generally performed approximately 8 weeks after completion of the planned neoadjuvant sequence, whether LCCRT alone or TNT. MRI-derived variables included clinical T stage, clinical nodal stage, maximum craniocaudal tumor length, distance from the anal verge, mesorectal fascia involvement, extramural venous invasion, mesorectal nodal involvement, and extramesorectal nodal involvement. Tumor length and distance from the anal verge were recorded in millimeters.

Clinical complete response was defined using combined digital rectal examination, endoscopy, and MRI criteria, including absence of residual tumor on examination, a flat white scar or telangiectasia without ulceration or nodularity on endoscopy, and MRI findings consistent with fibrosis without residual tumor signal. Clinical complete response was assigned by multidisciplinary consensus according to commonly used watch-and-wait criteria.

Patients with seemingly cCR were re-evaluated by the institutional multidisciplinary tumor board for possible nonoperative watch-and-wait management. Nonoperative surveillance included digital rectal examination, endoscopy, and carcinoembryonic antigen testing every 3–4 months during the first two years, pelvic MRI, CT chest/abdomen every 6 months during the first two years, and annual CT thereafter up to 5 years.

All MRI examinations were interpreted by dedicated radiologists with expertise in rectal cancer imaging. Radiologists performing restaging assessments were blinded to treatment strategy and did not know whether patients had received LCCRT or TNT.

### 2.3. Treatment Protocols

Treatment strategy was decided by our unit multidisciplinary tumor board based on baseline clinical status, performance status, comorbidities, tumor location, and MRI-defined risk features. TNT was generally considered for patients with higher-risk locally advanced rectal cancer features, including cT4 tumors, N2 nodal disease, extramural venous invasion, or threatened mesorectal fascia. Patients without these high-risk characteristics, or those considered less suitable for intensified systemic chemotherapy, were typically treated with standard LCCRT followed by surgery.

#### 2.3.1. Long-Course Chemoradiotherapy

Patients treated with LCCRT received pelvic radiotherapy to 45 Gy in 25–28 fractions over approximately four to five weeks, followed by a sequential boost to a total dose of 50–56 Gy in most patients. Radiotherapy was delivered using modern conformal techniques, including intensity-modulated radiotherapy (IMRT) or volumetric-modulated arc therapy (VMAT), with daily image guidance. The irradiation plan was individualized according to tumor extent, pelvic anatomy, and organ-at-risk constraints. Concurrent chemotherapy consisted predominantly of capecitabine-based regimens.

#### 2.3.2. Total Neoadjuvant Therapy

In the TNT cohort, long-course chemoradiotherapy was delivered similarly to the LCCRT protocol. Systemic chemotherapy was administered either before chemoradiotherapy as induction chemotherapy or after chemoradiotherapy as consolidation chemotherapy, according to multidisciplinary decision-making and institutional practice. Chemotherapy regimens included CAPOX, FOLFOX, or modified FOLFIRINOX. The choice of chemotherapy regimen was individualized by the multidisciplinary tumor board and medical oncology team according to patient age, performance status, comorbidities, renal function, anticipated treatment tolerance, and baseline tumor-risk profile. Modified FOLFIRINOX was reserved for selected fit patients with high-risk disease, whereas CAPOX and FOLFOX were more commonly used in routine practice. Patients in the TNT cohort received at least six cycles of systemic chemotherapy whenever clinically feasible.

#### 2.3.3. Surgical and Pathological Assessment

All surgical procedures were performed at our institution according to total mesorectal excision principles. The type of surgery and feasibility of sphincter preservation were determined by tumor location, response to neoadjuvant therapy, local anatomy, anticipated circumferential resection margin status, and multidisciplinary surgical planning. Pathological complete response was defined as the absence of viable residual tumor in the rectal wall and regional lymph nodes, corresponding to ypT0N0. Tumor regression was assessed using the Dworak grading system, with grade 4 indicating complete regression of the primary tumor.

### 2.4. Study Endpoints

The primary endpoints were clinical complete response and pathological complete response. Clinical complete response was assessed among all patients after neoadjuvant therapy, whereas pathological complete response was assessed only among patients who underwent surgical resection.

Secondary endpoints included radiologic tumor regression, nodal downstaging, conversion of mesorectal fascia involvement, conversion of extramural venous invasion status, surgical resection rate, sphincter preservation rate, reasons for nonoperative management, and overall survival.

Radiologic tumor regression was assessed using MRI-based change in tumor length between baseline and restaging MRI. MRI tumor-length response analyses were restricted to patients with available paired baseline and restaging measurements. Nodal, mesorectal fascia, and EMVI conversion were assessed among patients with baseline positive findings. Overall survival was defined as the time from diagnosis to death from any cause.

### 2.5. Statistical Analysis

Continuous variables were summarized as mean ± standard deviation or median with interquartile range, as appropriate, and compared using Student’s *t* test or the Mann–Whitney U test. Categorical variables were compared using Fisher’s test because of the relatively small sample size. All tests were two-sided, with *p* < 0.05 considered statistically significant.

To reduce treatment-selection bias, propensity score matching was performed using a logistic regression model including age, sex, clinical T stage, clinical nodal status, mesorectal and extramesorectal nodal involvement, mesorectal fascia status, and extramural venous invasion. Patients were matched 1:1 using nearest-neighbor matching without replacement. Covariate balance was evaluated using standardized mean differences, with values below 0.1 considered acceptable.

Associations between treatment strategy and complete response outcomes were evaluated using logistic regression models. Because of the limited number of cCR and pCR events, Firth penalized logistic regression was applied for adjusted analyses to reduce potential small-sample and rare-event bias. Covariates for adjusted models were selected a priori based on clinical relevance, observed baseline imbalance between treatment groups, and the limited number of outcome events. Accordingly, adjusted models included treatment group and baseline cT4 status. Baseline cT4 stage was included because it differed significantly between treatment groups and represents a clinically relevant marker of local tumor burden. Multivariable models were intentionally parsimonious to reduce the risk of overfitting.

Overall survival was calculated from diagnosis to death from any cause. Survival status was confirmed using hospital records and official governmental death registries, with administrative censoring on 1 January 2026. Median follow-up was estimated using the reverse Kaplan–Meier method. Survival probabilities were estimated using Kaplan–Meier curves and compared using the log-rank test. Restricted mean survival time up to 36 months was calculated with corresponding 95% confidence intervals.

Univariable and multivariable Cox proportional hazards regression analyses were used to evaluate predictors of overall survival, with results reported as hazard ratios and 95% confidence intervals. The proportional hazards assumption was assessed using Schoenfeld residuals. Sensitivity analyses were conducted for the multivariable survival model. Missing data were handled using complete-case analysis for each specific endpoint or model. Baseline tumor length had missing values in 8 of 110 patients, and MRI tumor-length response analyses were restricted to patients with available paired baseline and restaging MRI measurements. No imputation was performed.

All analyses were performed using R version 4.5.2. Because of the retrospective design, no formal sample size calculation was performed; the sample size was determined by the number of eligible patients treated during the study period.

## 3. Results

### 3.1. Patient Cohort

A total of 110 patients with locally advanced rectal cancer (stage II–III) were included, of whom 78 received LCCRT and 32 received TNT.

Baseline demographic and clinicopathologic characteristics are summarized in Table 1. Patients in the TNT cohort were significantly younger (61.8 ± 9.4 vs. 66.4 ± 10.7 years; *p* = 0.038) and had a higher proportion of cT4 tumors (40.6% vs. 19.2%; *p* = 0.014) than those in the LCCRT group. Tumor location, tumor length, MRF involvement, EMVI status, and clinical nodal stage were otherwise broadly comparable between groups.

### 3.2. Neoadjuvant Treatment Characteristics

Treatment characteristics are summarized in Table 2. In the TNT cohort, systemic chemotherapy consisted predominantly of oxaliplatin-based doublets, most commonly CAPOX followed by FOLFOX, while only one patient received mFOLFIRINOX. All patients in the LCCRT group received concurrent capecitabine-based chemotherapy. Radiotherapy boost doses were comparable between groups, with 50 Gy being the most frequently delivered total dose in both cohorts.

### 3.3. Tumor Response and Surgical Outcomes

Tumor response and surgical outcomes are summarized in Table 3 and Supplementary Tables S1–S4. Radiologic tumor response on restaging MRI was comparable between groups. Among patients with available paired MRI measurements, tumor length decreased in 48 of 56 LCCRT patients and in 15 of 19 TNT patients (85.7% vs. 78.9%; *p* = 0.742). Of those with tumor length reduction, shrinkage greater than 30% was observed in 30 of 48 LCCRT patients and 9 of 15 TNT patients (62.5% vs. 60.0%; *p* = 1.00).

**Figure 2:**
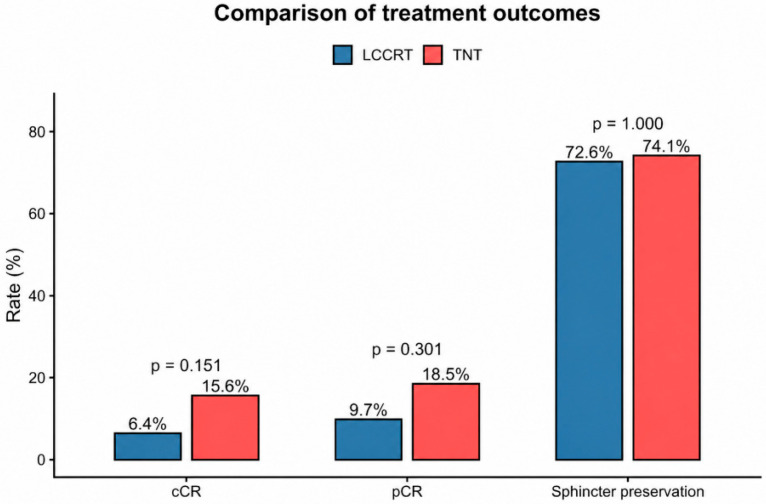
Comparison of treatment outcomes between long-course chemoradiotherapy and total neoadjuvant therapy.

Clinical complete response was achieved in 5 of 78 LCCRT patients and 5 of 32 TNT patients (6.4% vs. 15.6%; *p* = 0.151). Surgical resection was performed in 62 of 78 LCCRT patients and 27 of 32 TNT patients (79.5% vs. 84.4%; *p* = 0.790). Among operated patients, sphincter-preserving surgery was performed in 45 of 62 LCCRT patients and 20 of 27 TNT patients (72.6% vs. 74.1%; *p* = 1.00). Pathological complete response was observed in 6 of 62 resected LCCRT patients and 5 of 27 resected TNT patients (9.7% vs. 18.5%; *p* = 0.301). (Figure 2).

MRI-defined high-risk feature clearance was also similar between treatment groups. Mesorectal lymph node clearance occurred in 31 of 69 LCCRT patients and 13 of 32 TNT patients (44.9% vs. 40.6%; *p* = 1.00), while extramesorectal lymph node clearance occurred in 16 of 26 and 6 of 9 patients, respectively (61.5% vs. 66.7%; *p* = 1.00). Conversion from positive to negative mesorectal fascia status was observed in 19 of 39 LCCRT patients and 5 of 15 TNT patients (48.7% vs. 33.3%; *p* = 1.00), and EMVI clearance occurred in 7 of 13 and 3 of 8 patients, respectively (53.8% vs. 37.5%; *p* = 1.00).

Twenty-one patients did not undergo surgery, including 16 in the LCCRT group and 5 in the TNT group. The reasons for nonoperative management were disease progression, clinical complete response, refusal of surgery, or loss to follow-up, without a significant difference in distribution between treatment groups (*p* = 0.629). Overall, cCR and pCR were numerically higher after TNT, whereas MRI tumor-length response, MRI-defined high-risk feature clearance, surgical resection rate, and sphincter preservation were comparable between groups.

### 3.4. Survival Outcomes

**Figure 3:**
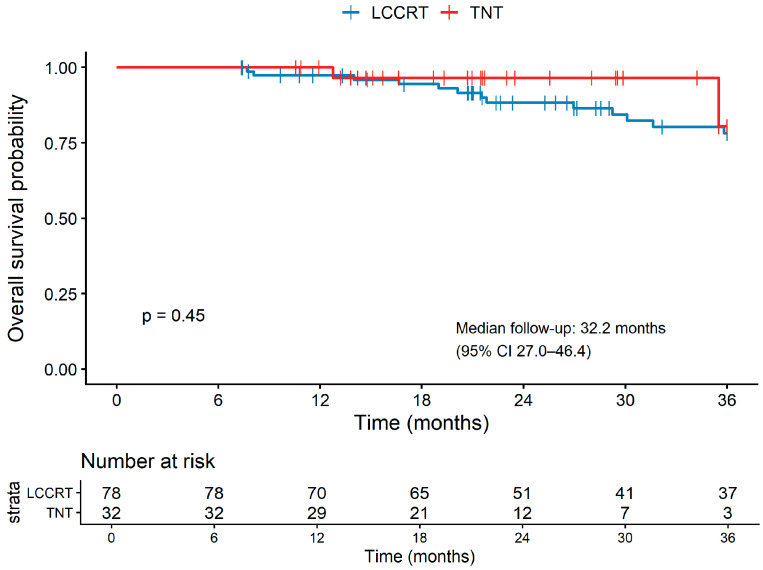
Kaplan–Meier curves for overall survival according to treatment strategy (TNT vs. LCCRT).

Survival outcomes are summarized in Table 4, Figure 3, and Supplementary Tables S5 and S6. A total of 19 deaths were recorded during follow-up, including 15 deaths within the 36-month administratively censored survival analysis. Kaplan–Meier curves showed substantial overlap between treatment groups, and no statistically significant difference in overall survival was observed between patients treated with TNT and those treated with LCCRT (log-rank *p* = 0.45). Restricted mean survival time up to 36 months was 35.12 months in the TNT group and 33.20 months in the LCCRT group, corresponding to a nonsignificant RMST difference of 1.92 months in favor of TNT (95% CI −0.28 to 4.11; *p* = 0.087).

In univariable Cox regression, baseline cT4 stage was the only variable significantly associated with worse 36-month overall survival (HR 3.15, 95% CI 1.17–8.44; *p* = 0.023), whereas treatment group, age, nodal status, EMVI, and MRF involvement were not significantly associated with survival (Supplementary Table S7). In a parsimonious multivariable Cox model adjusted for treatment group, age, and baseline cT4 status, TNT was not independently associated with 36-month overall survival (HR 0.46, 95% CI 0.09–2.25; *p* = 0.339). Baseline cT4 disease remained independently associated with worse survival (HR 3.42, 95% CI 1.20–9.75; *p* = 0.021). The proportional hazards assumption was not violated (global Schoenfeld test *p* = 0.69). Sensitivity analyses including additional MRI-defined baseline risk factors did not materially change these findings.

Overall, no statistically significant survival difference was observed between treatment strategies, while baseline cT4 disease was the only variable independently associated with worse 36-month overall survival.

### 3.5. Propensity Score Matched Analysis

Propensity score matching generated a matched cohort of 62 patients, including 31 patients treated with LCCRT and 31 treated with TNT (Supplementary Table S8). After matching, baseline comparability improved across the selected variables. In the matched cohort, clinical complete response occurred in 1 of 31 LCCRT patients and 5 of 31 TNT patients (3.2% vs. 16.1%; *p* = 0.195). Among resected matched patients, pathological complete response occurred in 1 of 26 LCCRT patients and 5 of 26 TNT patients (3.8% vs. 19.2%; *p* = 0.191) (Table 5 and Supplementary Table S9). Although cCR and pCR were numerically higher in the TNT group after matching, these differences did not reach statistical significance and should be interpreted as exploratory findings, limited by the small matched sample size and sparse number of events (Supplementary Figure S1). Adjusted and propensity score-matched analyses were performed to account for baseline imbalance between treatment groups and to evaluate whether the observed differences in response outcomes persisted after adjustment (Table 5, Supplementary Tables S10 and S11 and Figure S2). In multivariable Firth penalized logistic regression adjusted for treatment group and baseline cT4 stage, TNT was associated with higher adjusted odds of clinical complete response compared with LCCRT, although this association did not reach statistical significance (OR 3.25, 95% CI 0.85–12.57; *p* = 0.083). A similar nonsignificant trend was observed for pathological complete response (OR 2.21, 95% CI 0.59–8.03; *p* = 0.229). Baseline cT4 stage was not significantly associated with either cCR or pCR in these adjusted models. After matching and Firth penalized regression, TNT remained associated with numerically higher complete response estimates, but none of these associations reached statistical significance.

Sphincter preservation was evaluated among operated patients using multivariable logistic regression. TNT was not independently associated with sphincter-preserving surgery (OR 0.96, 95% CI 0.23–3.94; *p* = 0.957) (Table 6). Greater baseline distance from the anal verge was independently associated with a higher likelihood of sphincter preservation (OR 1.05 per mm, 95% CI 1.03–1.08; *p* < 0.001), whereas baseline mesorectal fascia involvement was associated with a lower likelihood of sphincter preservation (OR 0.22, 95% CI 0.06–0.82; *p* = 0.025). Sphincter preservation was associated with baseline anatomic factors rather than treatment strategy.

## 4. Discussion

In this real-world cohort of patients with locally advanced rectal cancer, TNT was preferentially administered to patients with more advanced baseline disease, including a significantly higher proportion of cT4 tumors. cCR and pCR rates were numerically higher after TNT than after LCCRT, but these differences did not reach statistical significance. MRI-based tumor-length response, high-risk MRI feature clearance, surgery rate, sphincter preservation, and short-term overall survival were broadly comparable between treatment strategies. These findings support the feasibility of TNT in routine clinical practice among higher-risk patients, but complete response differences should be interpreted cautiously given the retrospective design, baseline imbalance, and limited number of response events.

The higher prevalence of cT4 tumors in the TNT group likely reflects treatment-selection bias and evolving institutional practice patterns. Adjusted Firth penalized logistic regression and propensity score matching showed numerically higher complete response estimates with TNT, but confidence intervals were wide and statistical significance was not reached. Therefore, these findings should be considered exploratory and hypothesis-generating; treatment superiority cannot be inferred from this retrospective cohort.

Our findings are broadly consistent with randomized trials showing higher tumor response rates with TNT-based strategies compared with conventional chemoradiotherapy. RAPIDO and PRODIGE-23 reported higher pCR rates and improved disease-related outcomes with TNT-based treatment [8,9], while CAO/ARO/AIO-12 highlighted the relevance of chemotherapy sequencing within TNT strategies [10]. Systematic reviews and meta-analyses have similarly confirmed higher response rates with TNT, although improvements in long-term survival outcomes remain less consistent, and the optimal sequence of induction versus consolidation chemotherapy remains under investigation [11,12,13,14,15,28,29,30,31,32,33].

Recent real-world studies have also reported favorable response outcomes with TNT, although results remain heterogeneous because of differences in patient selection, treatment sequencing, chemotherapy regimen, radiotherapy approach, and response assessment. Şenocak Taşçı et al. reported higher pCR rates with TNT than with conventional chemoradiotherapy (21.8% vs. 2.9%), together with increased use of nonoperative management (16.9% vs. 0.9%) [34]. Ng et al. reported a pCR rate of 26.4% in a high-volume TNT cohort enriched for high-risk MRI features [25], while Zhou et al. observed increasing pCR rates from conventional CRT to short- and longer-course TNT strategies, although differences were attenuated after propensity score matching [35]. In this context, the pCR rate in our TNT cohort was lower than in some larger real-world series but remained within the range reported in routine practice, particularly considering the high-risk baseline profile, treatment heterogeneity, and limited sample size. Compared with previous real-world reports, the present study adds detailed paired baseline and restaging MRI assessment of MRF, EMVI, mesorectal nodal, and extramesorectal nodal clearance, while also integrating clinical, pathological, surgical, sphincter-preservation, survival, propensity-matched, and Firth-adjusted analyses in a single institutional cohort.

Several factors may explain the lower absolute pCR rate observed in our TNT cohort compared with randomized trials. In routine practice, response may be influenced by treatment heterogeneity, chemotherapy intensity, sequencing, and patient selection. In contrast to PRODIGE-23, where mFOLFIRINOX was systematically used [9], most patients in our TNT cohort received CAPOX or FOLFOX, with only a small proportion receiving triplet chemotherapy. Real-world and systematic evidence similarly suggests that TNT outcomes vary according to regimen and patient selection [25,28]. A further retrospective single-arm study of integrated intensified chemoradiation within a TNT framework also supported the feasibility and short-term efficacy of intensified neoadjuvant strategies in LARC patients with high-risk features [36]. In addition, the high-risk baseline profile of our cohort may have contributed to the lower absolute response rates, as larger tumors, more advanced T stage, and nodal involvement have been associated with reduced likelihood of pathological or clinical complete response after neoadjuvant therapy [37,38,39]. Therefore, the lower absolute cCR and pCR rates observed in this cohort may reflect the complexity of patients treated in routine practice rather than reduced efficacy of TNT itself.

MRI-based response assessment provided additional insight into treatment effects. High-resolution MRI is central to pretreatment evaluation of locally advanced rectal cancer because it assesses local tumor extent, mesorectal fascia involvement, nodal disease, and extramural venous invasion [20,22,24]. In this study, unidimensional MRI tumor-length reduction was similar between treatment groups, and approximately 60% of patients with tumor shrinkage in each group achieved a reduction greater than 30%. This finding illustrates the limited ability of linear measurements to serve as surrogates for treatment response. Post-neoadjuvant MRI interpretation is complex because residual abnormalities may reflect fibrosis, edema, mucinous change, inflammation, or residual viable tumor, which may not be adequately captured by tumor-length reduction alone [40,41]. Because rectal tumors are often irregularly shaped and difficult to measure reproducibly in a single plane, three-dimensional volumetric assessment may provide a more accurate estimate of tumor regression, although it is more time-consuming and less widely implemented in routine practice [40]. Diffusion-weighted imaging may improve differentiation between residual tumor and fibrosis, while radiomics-based multiparametric MRI approaches may further capture tumor heterogeneity and treatment-related biological changes [40,42]. Future studies incorporating standardized volumetric MRI, diffusion-weighted imaging/ADC analysis, and radiomics-based response assessment may provide a more comprehensive characterization of treatment response after TNT and LCCRT [42].

Clearance of MRI-defined high-risk features was also comparable between groups. Mesorectal lymph node, extramesorectal lymph node, mesorectal fascia, and EMVI clearance occurred in substantial proportions of patients after both treatment strategies, without statistically significant differences. The lack of a clear difference between groups may reflect limited sample size, baseline imbalance, or the fact that MRI-defined feature clearance and complete tumor response do not necessarily measure the same biological process. Achieving a negative circumferential resection margin remains one of the most important determinants of local control and long-term oncologic outcomes [2,3], while EMVI has been associated with systemic dissemination and adverse survival outcomes [21,23]. Therefore, lymph node downstaging, MRF clearance, EMVI regression, cCR, and pCR should be interpreted as related but distinct response endpoints.

Clinical complete response is clinically relevant because it may allow selected patients to undergo a structured watch-and-wait strategy, potentially avoiding rectal resection and its functional consequences. This may be particularly important for elderly or comorbid patients and those with ultra-low rectal tumors, in whom radical rectal surgery carries substantial functional or operative consequences [43]. The OPRA trial demonstrated that TNT-based organ preservation can be achieved while maintaining favorable oncologic outcomes [16,18,44], and earlier work by Habr-Gama, together with subsequent prospective and registry studies, supports nonoperative management in carefully selected patients with sustained cCR under strict surveillance [17,19,44,45,46,47,48,49]. In the present cohort, however, cCR remained infrequent, and the difference between TNT and LCCRT did not reach statistical significance.

Surgical outcomes were comparable between treatment strategies. Approximately 80% of patients in both groups underwent resection, and sphincter-preserving surgery was achieved in approximately three quarters of operated patients, without a significant difference between TNT and LCCRT. In multivariable analysis, sphincter preservation was not independently associated with treatment strategy but was primarily related to baseline anatomic factors, particularly distance from the anal verge and mesorectal fascia involvement. This is clinically intuitive, as sphincter preservation is strongly influenced by distal tumor extent, anticipated circumferential margin status, and local anatomy. The oncologic importance of total mesorectal excision quality and circumferential margin status is well established [1,2,3]. Importantly, TNT did not appear to compromise surgical feasibility in this real-world cohort.

Overall survival did not differ significantly between treatment groups during the observation period. Kaplan–Meier curves showed substantial overlap, and TNT was not independently associated with survival in multivariable Cox regression. Baseline cT4 stage remained significantly associated with worse overall survival, emphasizing the prognostic importance of local tumor burden. These survival findings should be considered preliminary and exploratory given the relatively short follow-up, particularly in the TNT cohort, and the limited number of events. Therefore, the absence of a statistically significant overall survival difference should not be interpreted as evidence of equivalent long-term oncologic outcomes. Randomized TNT trials have more consistently demonstrated improvements in pCR, disease-related treatment failure, or disease-free survival than early overall survival [8,9,10]. Longer follow-up and larger cohorts are required to determine whether response differences in routine practice translate into durable oncologic benefit.

This study has several limitations. First, its retrospective design introduces potential selection bias. Treatment allocation was not randomized, and TNT was preferentially administered to patients with more advanced baseline disease. Although propensity score matching and adjusted analyses were used to reduce baseline imbalance, residual confounding cannot be excluded. Second, the modest sample size and limited number of complete response events reduced statistical power, increasing the risk of type II error. Third, treatment heterogeneity within the TNT group may have influenced response, as patients received different chemotherapy regimens and sequencing approaches. In addition, although all patients received modern conformal radiotherapy using IMRT or VMAT, the institutional database did not contain sufficiently standardized dosimetric and planning parameters to allow robust comparison between techniques. Fourth, shorter follow-up in the TNT cohort limited interpretation of longer-term survival outcomes. Finally, this single-center study may not be directly generalizable to institutions with different patient populations, treatment protocols, or multidisciplinary decision-making practices.

Despite these limitations, the study has several strengths. It reflects real-world multidisciplinary management of locally advanced rectal cancer, including patients with high-risk baseline features who are often underrepresented in controlled trial populations. Detailed baseline and restaging MRI data allowed assessment of both tumor-length response and high-risk feature clearance. In addition, propensity score matching and Firth penalized logistic regression provided a cautious analytical framework for addressing baseline imbalance and sparse response events.

## 5. Conclusions

In this retrospective real-world cohort, TNT was preferentially used in patients with higher-risk locally advanced rectal cancer and showed numerically higher clinical and pathological complete response rates compared with LCCRT, although these differences were not statistically significant. MRI-based response, high-risk feature clearance, surgical outcomes, sphincter preservation, and short-term overall survival were broadly comparable between treatment groups. These findings support the feasibility of TNT in routine clinical practice, particularly among higher-risk patients, but remain exploratory and do not establish treatment superiority. Larger prospective studies with standardized TNT regimens and longer follow-up are needed to better define which patients derive the greatest benefit from intensified neoadjuvant treatment.

## Figures and Tables

**Table 1:** Baseline demographic and clinicopathologic characteristics.

Characteristic	LCCRT (n = 78)	TNT (n = 32)	*p*-Value
**Age, years**	66.4 ± 10.7	61.8 ± 9.4	0.038
**Sex**			0.60
Male	52 (66.7%)	23 (71.9%)	
Female	26 (33.3%)	9 (28.1%)	
**Comorbidities**			
Ischemic heart disease	22 (28.2%)	15 (46.9%)	0.060
Diabetes mellitus	16 (20.5%)	9 (28.1%)	0.40
Chronic kidney disease	3 (3.8%)	1 (3.1%)	>0.99
COPD	1 (1.3%)	1 (3.1%)	0.50
**Tumor stage**			
cT2–3	63 (80.8%)	18 (56.3%)	0.014
cT4	15 (19.2%)	13 (40.6%)	
**Clinical nodal stage**			0.20
cN0	7 (9.0%)	0 (0%)	
cN1–2	71 (91.0%)	31 (100%)	
**Baseline MRI features**			
Mesorectal lymph node positive	69 (88.5%)	32 (100%)	0.056
Extramesorectal lymph node positive	26 (33.3%)	9 (28.1%)	0.60
Mesorectal fascia involvement	39 (50.0%)	15 (46.9%)	0.80
Extramural venous invasion	13 (16.7%)	8 (25.0%)	0.30
**Tumor length,** mm (median [IQR])	53.0 [44.8–63.8]	57.5 [50.3–73.0]	0.062
**Tumor length cT4 stage,** mm (median [IQR])	60.0 [53.0–71.0]	70.0 [52.0–90.0]	0.489
**Tumor location**			0.80
<5 cm from anal verge	30 (38.5%)	12 (37.5%)	
5–10 cm	37 (47.4%)	14 (43.8%)	
>10 cm	11 (14.1%)	6 (18.8%)	

**Note:** Values are presented as mean ± standard deviation (SD) or number (percentage). COPD, chronic obstructive pulmonary disease; LCCRT, long-course chemoradiotherapy; TNT, total neoadjuvant therapy.

**Table 2:** Neoadjuvant treatment characteristics.

Treatment Characteristic	LCCRT (n = 78)	TNT (n = 32)
**Chemotherapy regimen**		
CAPOX	—	19 (59.4%)
FOLFOX	—	11 (34.4%)
mFOLFIRINOX	—	1 (3.1%)
Missing	—	1 (3.1%)
**Radiotherapy boost dose (Gy)**		
45	1 (1.3%)	—
50	51 (65.4%)	19 (59.4%)
54	12 (15.4%)	8 (25.0%)
56	14 (17.9%)	5 (15.6%)

**Note:** CAPOX, capecitabine plus oxaliplatin; FOLFOX, fluorouracil, leucovorin, and oxaliplatin; mFOLFIRINOX, modified fluorouracil, leucovorin, irinotecan, and oxaliplatin; Gy, gray.

**Table 3:** Tumor response, MRI feature clearance, and surgical outcomes.

Outcome	LCCRT	TNT	*p*-Value
Tumor length decreased on MRI	48/56 (85.7%)	15/19 (78.9%)	0.742
Tumor shrinkage > 30%	30/48 (62.5%)	9/15 (60.0%)	1.00
Clinical complete response	5/78 (6.4%)	5/32 (15.6%)	0.151
Underwent surgical resection	62/78 (79.5%)	27/32 (84.4%)	0.790
Sphincter-preserving surgery	45/62 (72.6%)	20/27 (74.1%)	1.00
Pathological complete response	6/62 (9.7%)	5/27 (18.5%)	0.301
Mesorectal lymph node clearance	31/69 (44.9%)	13/32 (40.6%)	1.00
Extramesorectal lymph node clearance	16/26 (61.5%)	6/9 (66.7%)	1.00
Mesorectal fascia clearance	19/39 (48.7%)	5/15 (33.3%)	1.00
EMVI clearance	7/13 (53.8%)	3/8 (37.5%)	1.00

**Note:** MRI response was calculated among patients with available paired baseline and restaging MRI tumor-length measurements. Tumor shrinkage > 30% was calculated among patients with tumor length decrease. Sphincter-preserving surgery and pathological complete response were calculated among operated/resected patients. EMVI, extramural venous invasion; LCCRT, long-course chemoradiotherapy; MRI, magnetic resonance imaging; TNT, total neoadjuvant therapy.

**Table 4:** Summary of 36-month overall survival analyses.

Outcome/Variable	Estimate	95% CI	*p*-Value
Log-rank comparison, TNT vs. LCCRT	—	—	0.45
RMST difference, TNT − LCCRT, months	1.92	−0.28 to 4.11	0.087
TNT vs. LCCRT	HR 0.46	0.09–2.25	0.339
Age, per year	HR 1.04	0.99–1.10	0.109
cT4 vs. non-cT4	HR 3.42	1.20–9.75	**0.021**

**Note:** Overall survival was administratively censored at 36 months. RMST, restricted mean survival time; HR, hazard ratio; CI, confidence interval; TNT, total neoadjuvant therapy; LCCRT, long-course chemoradiotherapy.

**Table 5:** Adjusted and propensity score-matched analyses of complete response.

Analysis	Outcome/Variable	Estimate	95% CI	*p*-Value
Propensity score matching	cCR, LCCRT vs. TNT	3.2% vs. 16.1%	—	0.195
Propensity score matching	pCR, LCCRT vs. TNT	3.8% vs. 19.2%	—	0.191
Firth logistic regression	TNT vs. LCCRT for cCR	OR 3.25	0.85–12.57	0.083
Firth logistic regression	cT4 stage for cCR	OR 0.63	0.11–2.61	0.545
Firth logistic regression	TNT vs. LCCRT for pCR	OR 2.21	0.59–8.03	0.229
Firth logistic regression	cT4 stage for pCR	OR 0.93	0.20–3.47	0.913

**Note:** Firth penalized logistic regression models were adjusted for treatment group and baseline cT4 stage. Propensity score matching generated a matched cohort of 31 LCCRT and 31 TNT patients. pCR was calculated among resected matched patients. cCR, clinical complete response; pCR, pathological complete response; OR, odds ratio; CI, confidence interval; LCCRT, long-course chemoradiotherapy; TNT, total neoadjuvant therapy.

**Table 6:** Multivariable logistic regression for sphincter-preserving surgery.

Variable	OR	95% CI	*p*-Value
TNT vs. LCCRT	0.96	0.23–3.94	0.957
Age, per year	0.98	0.92–1.04	0.452
Baseline distance from anal verge, per mm	1.05	1.03–1.08	<0.001
Baseline MRF involvement	0.22	0.06–0.82	0.025

**Note:** Sphincter-preservation analysis was restricted to operated patients; LCCRT, long-course chemoradiotherapy; TNT, total neoadjuvant therapy; OR, odds ratio; CI, confidence interval; MRF, mesorectal fascia.

## Data Availability

The original contributions presented in this study are included in the article/Supplementary Material. Further inquiries can be directed to the corresponding author.

## Supplementary Materials

The following supporting information can be downloaded at https://www.mdpi.com/article/10.3390/medsci14030393/s1, Supplementary Table S1. Detailed radiologic tumor response on restaging MRI; Supplementary Table S2. MRI-defined high-risk feature conversion; Supplementary Table S3. Reasons for nonoperative management; Supplementary Table S4. Subgroup analyses; Supplementary Table S5. Kaplan–Meier survival summary; Supplementary Table S6. Restricted mean survival time comparison; Supplementary Table S7. Univariable Cox regression analysis; Supplementary Table S8. Propensity score–matched baseline characteristics; Supplementary Table S9. Propensity score–matched outcomes; Supplementary Table S10. Firth penalized logistic regression for cCR; Supplementary Table S11. Firth penalized logistic regression for pCR; Supplementary Figure S1. Covariate balance before and after propensity score matching; Supplementary Figure S2. Forest plot of Firth penalized logistic regression results. The primary models were adjusted for treatment group and baseline cT4 stage; matched-cohort estimates were exploratory and should be interpreted cautiously because of sparse events.

## Abbreviations

The following abbreviations are used in this manuscript:

CAPOX	Capecitabine and Oxaliplatin
CEA	Carcinoembryonic Antigen
cCR	Clinical Complete Response
CI	Confidence Interval
cN	Clinical Nodal Stage
COPD	Chronic Obstructive Pulmonary Disease
CRM	Circumferential Resection Margin
CT	Computed Tomography
cT	Clinical Tumor Stage
cT4	Clinical T4 Stage
DWI	Diffusion-Weighted Imaging
EMVI	Extramural Venous Invasion
FOLFOX	Fluorouracil, Leucovorin, and Oxaliplatin
HR	Hazard Ratio
IMRT	Intensity-Modulated Radiotherapy
IQR	Interquartile Range
LARC	Locally Advanced Rectal Cancer
LCCRT	Long-Course Chemoradiotherapy
mFOLFIRINOX	Modified Fluorouracil, Leucovorin, Irinotecan, and Oxaliplatin
MRI	Magnetic Resonance Imaging
MRF	Mesorectal Fascia
OR	Odds Ratio
pCR	Pathological Complete Response
RMST	Restricted Mean Survival Time
SD	Standard Deviation
TME	Total Mesorectal Excision
TNT	Total Neoadjuvant Therapy
VMAT	Volumetric-Modulated Arc Therapy
ypT0N0	Absence of viable residual tumor in the rectal wall and regional lymph nodes (Pathological Complete Response)
