# Supplementary material for: Total Neoadjuvant Therapy Versus Long-Course Chemoradiotherapy in Locally Advanced Rectal Cancer: Real-World Tumor Response and Clinical Outcomes
Source: Med Sci (Basel). 2026 Jul 14;14(3):393. doi: 10.3390/medsci14030393 (PMC13414229; doi:10.3390/medsci14030393)
Supplement: Supplementary file 1 [file medsci-14-00393-s001.zip › medsci-4416538-supplementary.pdf]

Supplementary Table S1. Detailed radiologic tumor response on restaging MRI

Radiologic response	LCCRT (n=56)	TNT (n=19)	p-value
Tumor length decreased	48 (85.7%)	15 (78.9%)	
Tumor length increased	5 (8.9%)	2 (10.5%)	
Tumor length unchanged	3 (5.4%)	2 (10.5%)	0.742
	**LCCRT (n=48)**	**TNT (n=15)**	
Tumor shrinkage >30% †	30 (62.5%)	9 (60.0%)	1.00

Note: Percentages for tumor length change were calculated among patients with available paired baseline and restaging MRI tumor-length measurements; †Percentages for tumor shrinkage >30% were calculated among patients with tumor length decrease; MRI, magnetic resonance imaging.

Supplementary Table S2. MRI-defined high-risk feature conversion

MRI feature	LCCRT	TNT	p-value
Mesorectal lymph node clearance	31/69 (44.9%)	13/32 (40.6%)	1.00
Extramesorectal lymph node clearance	16/26 (61.5%)	6/9 (66.7%)	1.00
Mesorectal fascia clearance	19/39 (48.7%)	5/15 (33.3%)	1.00
Extramural venous invasion clearance	7/13 (53.8%)	3/8 (37.5%)	1.00

Supplementary Table S3. Reasons for nonoperative management

Reason	LCCRT (n = 16)	TNT (n = 5)
Clinical complete response	4 (25.0%)	2 (40.0%)
Disease progression	6 (37.5%)	1 (20.0%)
Refused surgery	3 (18.8%)	0
Lost to follow-up	3 (18.8%)	2 (40.0%)

Note: Percentages represent proportions within each treatment group. LCCRT, long-course chemoradiotherapy; TNT, total neoadjuvant therapy.

Supplementary Table S4. Subgroup analyses

Subgroup	LCCRT	TNT	p-value

**pCR among baseline cT4 tumors**	1/12 (8.3%)	2/11 (18.2%)	0.590
**cCR among tumors <5 cm**	4/30 (13.3%)	3/12 (25.0%)	0.387

Note: pCR, pathological complete response; cCR, clinical complete response. Analyses were restricted to patients within the specified subgroup categories.

Supplementary Table S5. Kaplan–Meier survival summary

Treatment group	N	Events	Restricted mean survival (months)	Standard error
LCCRT	78	13	33.20	0.79
TNT	32	2	35.12	0.79

Note: Survival estimates were administratively censored at 36 months; therefore, only deaths occurring within 36 months were included in the restricted mean survival time analysis.

Supplementary Table S6. Restricted mean survival time comparison

Comparison	RMST difference (months)	95% CI	p-value
**TNT − LCCRT**	1.92	−0.28 to 4.11	0.087

Note: RMST, restricted mean survival time; CI, confidence interval. RMST was calculated up to 36 months.

Supplementary Table S7. Univariable Cox regression analysis

Variable	Hazard Ratio (HR)	95% CI	p-value
TNT vs LCCRT	0.52	0.12–2.20	0.37
Age (per year)	1.04	0.99–1.09	0.12
cT4 vs non-cT4	3.15	1.17–8.44	**0.023**
cN2 vs cN0–1	1.41	0.48–4.15	0.53
EMVI positive	0.93	0.27–3.19	0.91
MRF positive	0.67	0.24–1.91	0.46

Note: HR, hazard ratio; CI, confidence interval; TNT, total neoadjuvant therapy; LCCRT, long-course chemoradiotherapy; EMVI, extramural venous invasion; MRF, mesorectal fascia.

Supplementary Table S8. Propensity score–matched baseline characteristics

Characteristic	LCCRT (n = 31)	TNT (n = 31)	SMD

Age, years	64.94 ± 10.94	62.06 ± 9.45	0.281
Female sex	8 (25.8%)	9 (29.0%)	0.072
cT4 stage	10 (32.3%)	13 (41.9%)	0.198
cN1–2 stage	31 (100.0%)	31 (100.0%)	<0.001
Mesorectal lymph node positive	31 (100.0%)	31 (100.0%)	<0.001
Extramesorectal lymph node positive	7 (22.6%)	9 (29.0%)	0.145
Mesorectal fascia involvement	12 (38.7%)	15 (48.4%)	0.193
Extramural venous invasion	2 (6.5%)	8 (25.8%)	0.537
Tumor location <5 cm	10 (32.3%)	11 (35.5%)	
Tumor location 5–10 cm	17 (54.8%)	14 (45.2%)	
Tumor location >10 cm	4 (12.9%)	6 (19.4%)	0.220†

Note: Values are presented as mean ± SD or number (percentage). SMD, standardized mean difference; LCCRT, long-course chemoradiotherapy; TNT, total neoadjuvant therapy. †Standardized mean difference shown for the overall tumor-location variable.

Supplementary Table S9. Propensity score–matched outcomes

Outcome	LCCRT (n=31)	TNT (n=31)	p-value
Clinical complete response	1 (3.2%)	5 (16.1%)	0.195
Pathological complete response*	1/26 (3.8%)	5/26 (19.2%)	0.191

Note: Percentages represent proportions within each treatment group. *pCR calculated among resected matched patients with available pathology data.

Supplementary Table S10. Firth penalized logistic regression for cCR

Variable	Adjusted odds ratio (95% CI)	p-value
TNT vs LCCRT	3.25 (0.85–12.57)	0.083
cT4 stage	0.63 (0.11–2.61)	0.545

Note: OR, odds ratio; CI, confidence interval; TNT, total neoadjuvant therapy; LCCRT, long-course chemoradiotherapy. Models were adjusted for treatment group and baseline cT4 stage.

Supplementary Table S11. Firth penalized logistic regression for pCR

Variable	Adjusted odds ratio (95% CI)	p-value
TNT vs LCCRT	2.21 (0.59–8.03)	0.229
cT4 stage	0.93 (0.20–3.47)	0.913

Note: OR, odds ratio; CI, confidence interval; TNT, total neoadjuvant therapy; LCCRT, long-course chemoradiotherapy. Models were adjusted for treatment group and baseline cT4 stage.

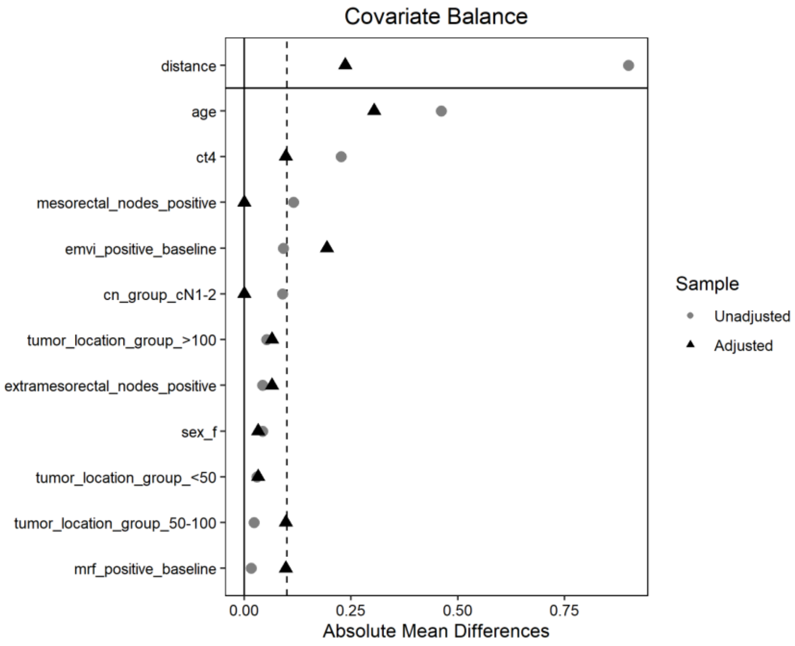

Supplementary Figure S1. Covariate balance before and after propensity score matching

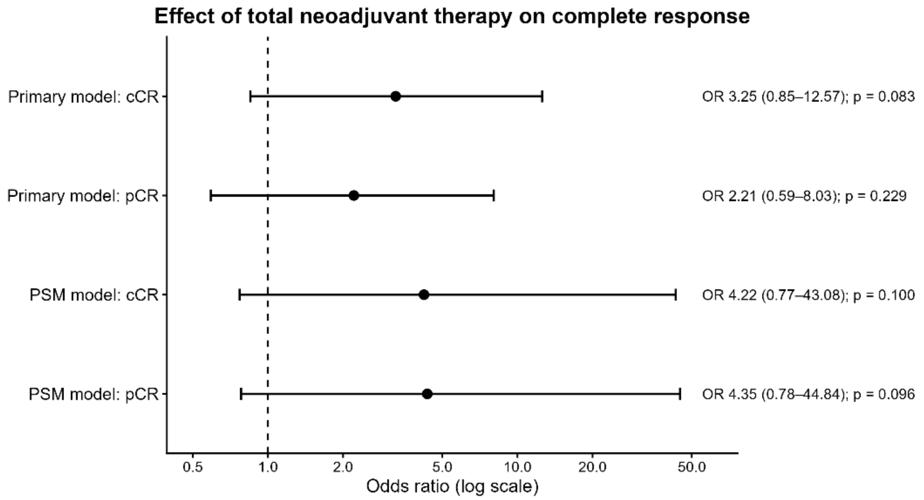

Supplementary Figure S2. Forest plot of Firth penalized logistic regression results. The primary models were adjusted for treatment group and baseline cT4 stage; matched-cohort estimates were exploratory and should be interpreted cautiously because of sparse events.
